# Does major pathological response after neoadjuvant Immunotherapy in resectable nonsmall-cell lung cancers predict prognosis? A systematic review and meta-analysis

**DOI:** 10.1097/JS9.0000000000000496

**Published:** 2023-05-26

**Authors:** Yujia Chen, Jianjun Qin, Yajing Wu, Qiang Lin, Jianing Wang, Wei Zhang, Fei Liang, Zhouguang Hui, Min Zhao, Jun Wang

**Affiliations:** a Department of Radiation Oncology, the Fourth Hospital of Hebei Medical University, Hebei Clinical Research Center for Radiation Oncology; b Department of Oncology, the First Hospital of Hebei Medical University, Shijiazhuang; c Department of Oncology, North China Petroleum Bureau General Hospital, Hebei Medical University, Renqiu; d Department of Biostatistics, Zhongshan Hospital, Fudan University, Shanghai; e Department of VIP Medical Services & Radiation Oncology; f Department of Thoracic Surgery, National Cancer Center/National Clinical Research Center for Cancer/Cancer Hospital, Chinese Academy of Medical Sciences and Peking Union Medical College, Beijing, China

**Keywords:** immunotherapy, major pathological response, neoadjuvant, nonsmall-cell lung cancer, survival

## Abstract

**Objective:**

Overall survival is the gold-standard outcome measure for phase 3 trials, but the need for a long follow-up period can delay the translation of potentially effective treatment to clinical practice. The validity of major pathological response (MPR) as a surrogate of survival for non small cell lung cancer (NSCLC) after neoadjuvant immunotherapy remains unclear.

**Methods:**

Eligibility was resectable stage I–III NSCLC and delivery of PD-1/PD-L1/CTLA-4 inhibitors prior to resection; other forms/modalities of neoadjuvant and/or adjuvant therapies were allowed. Statistics utilized the Mantel–Haenszel fixed-effect or random-effect model depending on the heterogeneity (*I* ^2^).

**Results:**

Fifty-three trials (seven randomized, 29 prospective nonrandomized, 17 retrospective) were identified. The pooled rate of MPR was 53.8%. Compared to neoadjuvant chemotherapy, neoadjuvant chemo-immunotherapy achieved higher MPR (OR 6.19, 4.39–8.74, *P*<0.00001). MPR was associated with improved disease-free survival/progression-free survival/event-free survival (HR 0.28, 0.10–0.79, *P*=0.02) and overall survival (HR 0.80, 0.72–0.88, *P*<0.0001). Patients with stage III (vs I/II) and PD-L1 ≥1% (vs <1%) more likely achieved MPR (OR 1.66,1.02–2.70, *P*=0.04; OR 2.21,1.28–3.82, *P*=0.004).

**Conclusions:**

The findings of this meta-analysis suggest that neoadjuvant chemo-immunotherapy achieved higher MPR in NSCLC patients, and increased MPR might be associated with survival benefits treated with neoadjuvant immunotherapy. It appears that the MPR may serve as a surrogate endpoint of survival to evaluate neoadjuvant immunotherapy.

## Introduction

Highlights

- **Question**: Does major pathological response (MPR) after neoadjuvant Immunotherapy in resectable nonsmall-cell lung cancers predict prognosis?
- **Findings**: Neoadjuvant immunotherapy could improve pathological responses obviously, as evidenced by a 53.4% pooled MPR rate. Neoadjuvant chemo-immunotherapy yielded promising efficacy, with an increased MPR rate compared with chemotherapy alone. MPR were shown to be associated with improved OS and disease-free survival/progression-free survival/event-free survival, and patients with stage III (vs I/II) or PD-L1 ≥1% (vs PD-L1<1%) were significantly more likely to achieve MPR.
- **Meaning**: The findings of this meta-analysis suggest that increased MPR may associate with survival benefits in NSCLC patients treated with neoadjuvant immunotherapy. The MPR may serve as a surrogate endpoint of OS to evaluate neoadjuvant immunotherapy. Our data can be provided.

Lung cancer is one of the most common and deadly cancers in the world^1^. Surgical resection remains the mainstay of treatment for early-stage and locally advanced nonsmall cell lung cancer (NSCLC). However, even with early-stage disease, 30–55% of patients with NSCLC develop recurrence and die of their disease despite curative resection^2–5^.

A meta-analysis of the NSCLC showed that adding chemotherapy for the neoadjuvant management could get a small gain in survival of 5% at 5 years^6^. Immune checkpoint inhibitors (ICIs) targeting the PD‐1/PD‐L1 axis, either as monotherapy or in combination with chemotherapy, are now the cornerstone of the treatment of metastatic NSCLC. Multiple phase 2 trials of neoadjuvant immunotherapy have shown encouraging outcomes that ICI alone or combined chemotherapy, effectively reduce the size of locally advanced tumors and improve their pathological regression^7^. The major pathological response (MPR), defined as 10% or less viable tumor, is in the range of 19–45%with single agent in the neoadjuvant setting, and fluctuates within 33–83% when combined with chemotherapy^8^. Recently, neoadjuvant nivolumab plus chemotherapy showed statistically significant longer event-free survival (EFS), better pathological complete response (pCR) rate and MPR rate compared with chemotherapy alone in the phase III CheckMate-816 trial^9^.

Although overall survival (OS) is the gold-standard outcome measure for phase 3 trials, the need for a long follow-up period can delay the translation of potentially effective treatment to clinical practice. MPR as a candidate surrogate endpoint to rapidly evaluate the clinical efficacy of neoadjuvant chemotherapy (nCT) has also been advocated. Weissferdt *et al*.^10^ identified 151 NSCLC patients who had been treated with nCT followed by complete surgical resection from 2008 to 2012. The results revealed that MPR was associated with long-term OS on multivariable analysis (HR=2.68, *P*=0.01). Hellman *et al*.^11^ proposed that MPR was strongly associated with improved survival, reflected the treatment impact and captured the magnitude of the treatment benefit on survival. So far, the evidence-based validity of MPR has not been demonstrated in the immunotherapy era.

Herein, we performed a systematic review and meta-analysis to estimate the validity of MPR as a surrogate of survival after neoadjuvant immunotherapy.

## Methods

### Systematic review

This study was registered at the PROSPERO database. AMSTAR 2, Supplemental Digital Content 1, http://links.lww.com/JS9/A615 and PRISMA, Supplemental Digital Content 2, http://links.lww.com/JS9/A616, Supplemental Digital Content 3, http://links.lww.com/JS9/A617 were used to evaluate methodological and reporting quality^12,13^. A systematic literature review was performed in MEDLINE, CENTRAL, EMBASE, as well as the proceedings of the American Association for Cancer Research (AACR), the European Lung Cancer Congress (ELCC), the American Society of Clinical Oncology (ASCO), the American Society for Radiation Oncology (ASTRO), and the International Association for the Study of Lung Cancer (IASLC) annual meetings. We additionally reviewed the reference lists of included publications along with relevant review articles retrieved from the electronic searches to identify other potentially relevant studies that could have been missed. A complete list of the search strategies for each database is provided in Supplemental Tables 1–3, Supplemental Digital Content 4, http://links.lww.com/JS9/A618.

PRISMA, Supplemental Digital Content 2, http://links.lww.com/JS9/A616 and MOOSE guidelines were followed as shown in Supplemental Tables 4–5, Supplemental Digital Content 5, http://links.lww.com/JS9/A619. The inclusion criteria of publications were defined according to the PICOD criteria, which was listed as follows. P: resectable NSCLC and confirmation of NSCLC with histopathology; I/E: neoadjuvant ICIs, including PD-1/PD-L1 inhibitors and CTLA-4 inhibitors, either combined with chemotherapy, radiotherapy, or lack thereof; C: either no control group (i.e. single-arm study); or nCT; O: MPR, disease-free survival (DFS), progression-free survival (PFS), EFS, recurrence-free survival (RFS), OS; D: randomized controlled trials, prospective nonrandomized trials, or observational (retrospective) studies. Searches did not have date restrictions and included articles in the English language that were published/presented through 12 October 2022.

More specifically, studies were eligible if they met the following inclusion criteria: histopathologically-confirmed stage I–III NSCLC with intent to perform an oncologic-quality/curative-intent resection (regardless of the proportion that eventually underwent resection); delivery of PD-1, PD-L1, or CTLA-4 inhibitors (regardless of dosing or cycles) prior to resection (nCT and/or radiotherapy, or any type of adjuvant therapy (or lack thereof), was allowed); sufficient data for quantitative meta-analysis for at least one outcome measure listed above (if the study pertained to a heterogeneous cohort, outcomes for the eligible population as defined above had to have been separately reported).

Other exclusion criteria were as follows: delivery of other treatment regimens/approaches prior to neoadjuvant therapy; meta-analyses, reviews, surveys, letters, case reports, and book chapters; studies based on the National Cancer Database or the Surveillance, Epidemiology, and End Results database, as these do not record the specific ICI agent; studies involving nonhuman subjects; and incomplete studies.

If a trial had been updated, we included only the publication with the most complete data. The data was reviewed by two independent authors (Y.J.W. and Q.L.) and validated with another two (J.W. and J.Y.C.) until consensus was reached. If important data in the included studies were missing, contacting the authors of the original publications was considered.

### Data extraction

From each study, extracted data included the first author’s name, study year, study design, baseline characteristics, neoadjuvant treatment regimen(s), histology, number of patients, MPR, PFS/DFS/EFS/RFS, and OS. Of note, for purposes of this meta-analysis, PFS/DFS/EFS/RFS were used interchangeably given the similar semantics and methodologies used to calculate these outcomes.

Different articles defined MPR and PCR differently; 33 articles reported that MPR contained PCR, 7 articles reported that MPR did not contain PCR, and 13 articles did not specify whether MPR contained PCR. We added the MPR given in the article with PCR as the MPR for this study in response to these seven papers where the MPR did not include PCR. We contacted the original author by e-mail to clarify the relationship between MPR and PCR in response to these 13 articles that did not specify whether the MPR contained PCR. Of these, four articles had explicitly stated that the MPR contained PCR, and two articles’ authors had not responded to the e-mail. However, seven articles in which we were unable to determine the author’s e-mail were therefore unable to contact the author.

### Evaluation of quality and bias

According to the Cochrane-Handbook for Systematic Reviews of Interventions, two authors independently assessed the methodological quality of the included randomized studies using the Cochrane Collaboration’s ‘Risk of Bias’ tool (Supplemental Figs. S1–2, Supplemental Digital Content 6, http://links.lww.com/JS9/A620, Supplemental Digital Content 7, http://links.lww.com/JS9/A621). The following domains were assessed: random sequence generation, allocation concealment, blinding of participants and personnel, blinding of outcome assessment (assessed separately for self-reported and objectively assessed outcomes), incomplete outcome data, selective reporting, and other sources of bias (specifically, baseline imbalance). Each item was rated as at ‘low risk’, ‘unclear risk’, or ‘high risk’ of bias. All review authors participated in resolving any discrepancies until a consensus was reached. For nonrandomized studies, the risk of bias was assessed by the MINORS score (Supplemental Table 6, Supplemental Digital Content 8, http://links.lww.com/JS9/A622). The items were scored 0 (not reported), one (reported but inadequate), or two (reported and adequate). The global ideal score was 16 for noncomparative studies and 24 for comparative studies.

Publication bias was examined by means of constructing funnel plots. Begg’s test were conducted to analyze the publication bias of all outcomes.

### Statistical analysis

The Review Manager (Rev Man) (version 5.4, provided by the Cochrane collaboration website at www.cochrane-handbook.org) software was used to evaluate publication bias, generate funnel plots and prediction intervals (PIs), evaluate for heterogeneity, as well as conduct the meta-analysis.

We assessed heterogeneity among trials using the *χ* ^2^-test for heterogeneity (with a 10% level of statistical significance) and the *I* ^2^ statistic. Data was pooled using the Mantel–Haenszel fixed-effect model if there was no significant heterogeneity (*I* ^2^<50%). If there was significant heterogeneity (*I* ^2^≥50%) the random-effect model was employed. Data regarding MPR was expressed as ORs with 95% CIs. For time-to-event data (DFS, PFS, EFS, RFS, OS), these were expressed as HRs with 95% CIs; additionally, the ORs or HRs were integrated to obtain the logHR or logOR and standard error (the inverse variance method was used to calculate the combined statistics). To quantify the association between DFS/PFS/EFS/RFS/OS and MPR, classical pairwise meta-analysis was conducted using a frequentist framework.

R software (version 4.2.0) was used to provide pooled estimates of the MPR of the single-group studies. The Meta-analysis for R (metafor) package (version 3.4-0) and the General Package for Meta-Analysis (meta) (version 5.2-0) were used to perform the random or fixed effects meta-analyses, tests for heterogeneity (*I* ^2^ and τ), generation of PIs, generation of funnel plots, and tests for publication bias (τ, which is the SD of the random-effect, to quantify study heterogeneity, was calculated using an arcsine transformation, with the value ranging from 0 to π; an inverse transform, (sin[τ/2])^2^, was used to express τ as a percentage in the article). The angular transformation was used and a 0.5 continuity correction was applied for studies with an event probability of 0 or 1. In addition, the restricted maximum likelihood method and the Knapp–Hartung adjustment were used. Weighted random-effect models and weighted fixed-effect models were used to determine an overall summary estimate for each outcome measure and were depicted on a forest plot with its corresponding 95% CI and associated 95% PIs. PIs were included because they were particularly insightful in this setting, with a 95% PI providing a prediction region for a single future study. The R code used to generate each of these analyses is provided in the Supplement 3, Supplemental Digital Content 9, http://links.lww.com/JS9/A623.

A sensitivity analysis was performed using Stata v12.0 according to the ‘leave-one-out’ method, which was used to determine the impact of each individual study on the overall results by removing each study. Each estimate value and its upper and lower CIs represented the HR or OR after the individual study was removed. The critical value of OR was 1 and the critical value of HR was 0. After removing any individual study, if the new HR or OR value was consistent with the original HR or OR value on the same side of the critical value, it was considered to verify the robustness of the particular outcome parameter.

## Results

### Literature review

**Figure 1:**
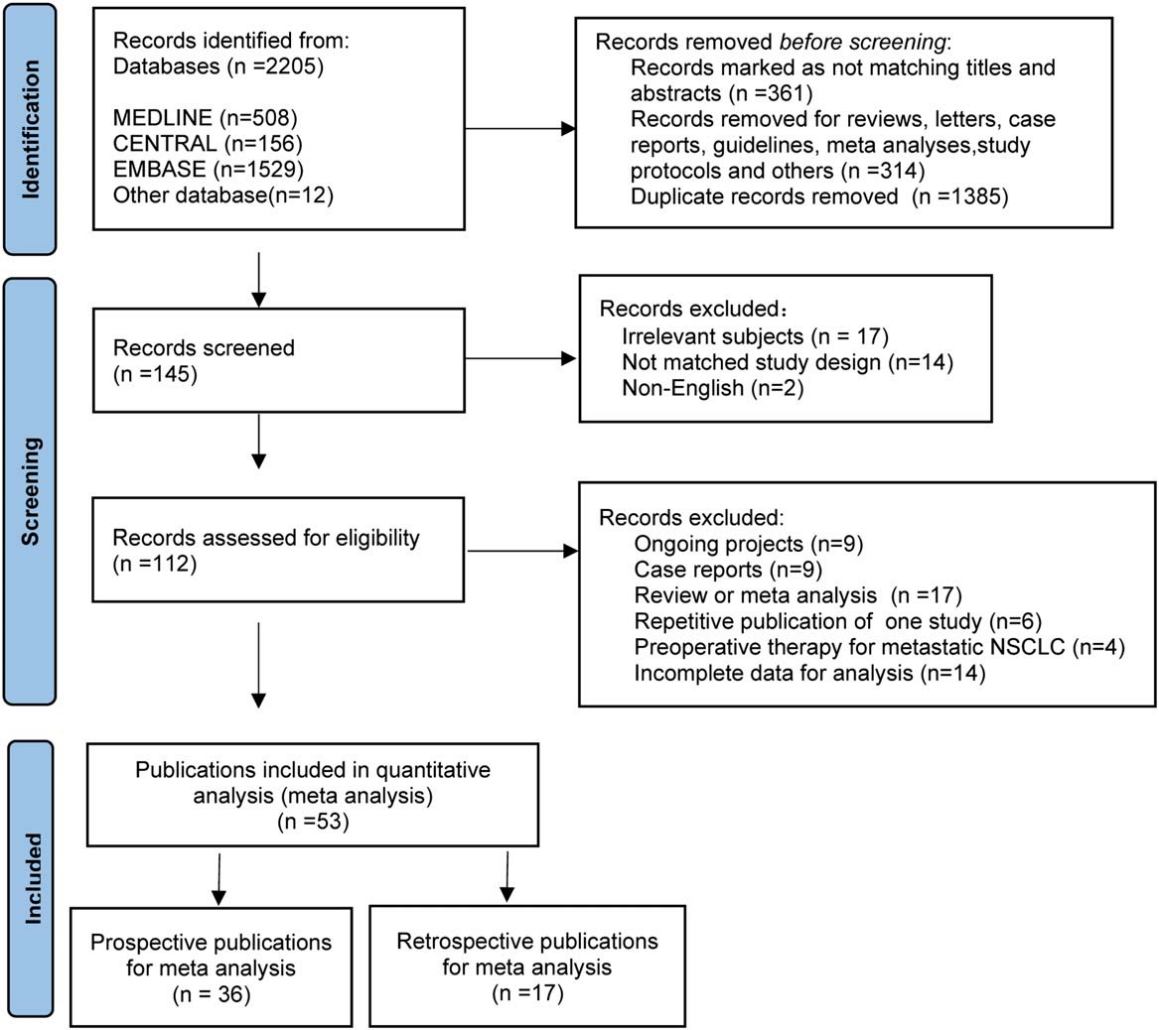
PRISMA flowchart of study selection.

Fifty-three articles met the criteria for this meta-analysis (Fig. 1), which included 7 randomized trials, 29 prospective nonrandomized trials, and 17 retrospective studies. There were nine studies that compared neoadjuvant chemo-immunotherapy (nCIT) versus nCT alone. Table 1 displayed pertinent details of each study^9,14–65^; patients were most commonly stage III and received 2–4 cycles of a variety of ICIs.

**Table 1:** General information from the included studies.

											OS	EFS/DFS/PFS						
Trial	Trial type	No. of patients,n	Resectable patients, %(n)	Histology	Clinical stage	R0% (*n*)	MPR% (n)	ORR% (*n*)	DCR% (*n*)	pCR% (*n*)	Survival (median/%)	Survival (median/%)	Neoadjuvant regimen	Neoadjuvant cycle	Adjuvant IO	≥G3 TRAEs%(n)	≥G3 SRAEs%(n)	Fatal AEs(n)
Forde PM et al, 2022 ^9^	randomised, controlled, phase III	179	149 (83.2%)	NSCLC	IB-IIIA	83.2% (124/149)	36.9% (66/179)	53.6% (96/179)	92.7% (166/179)	24% (43/179)	NR (median)	31.6 months (median),76.1% (1-year)63.8% (2-year)	nivolumab+Chemo	3	--	33.5%	--	5
		179	135 (75.4%)			77.8% (105/135)	8.9% (16/179)	37.4% (67/179)	86.6% (155/179)	2.2% (4/179)	NR (median)	20.8 months (median) 63.4% (1-year)45.3% (2-year)	Chemo		**--**	36.9%	--	4
Cascone, T. et al, 2021 ^14^	randomized, phase II	23	22 (95.7%)	SCC(39%), ASCC(2%), AC(59%)	I-IIIA	100% (22/22)	22% (5/23)	22% (5/23)	87% (20/23)	9% (2/22)	NR (median)	NR (median)	nivolumab	3	--	13%(3/23)	--	1
		21	17 (81.0%)			100% (17/17)	38% (8/21)	19% (4/21)	81% (17/21)	29% (6/21)	NR (median)	NR (median)	nivolumab+ipilimumab	3	--	10%(2/21)	--	0
Provencio et al.2022 ^15^	randomized, phase II	57	53 (93.0%)	NSCLC	IIIA-B	92.5%49/53	52% (30/57)	74% (42/57)	–	36.2% (21/57)	84.7% (2-year)	66.6% (2-year)	Nivolumab+Chemo	3	6 mo	24.6%(14/57)		0
		29	20 (69.0%)			65.0% (13/20)	14% (4/29)	48% (14/29)		6.8% (2/29)	63.4% (2-year)	42.3% (2-year)	Chemo	3		10.3%(3/29)		
Feng, Y et al, 2021^16^	randomized, phase II	8	8 (100.0%)	SCC(90.5%),Non-SCC(9.5%)	IIA-IIIB	–	50% (4/8)	87.5% (7/8)	100% (8/8	37.5% (3/8)	–	–	pembrolizumab or toripalimab+Chemo	2	--	12.5%(1/8)	--	0
		13	13 (100.0%)			–	38.46% (5/13)	46.15% (6/13)	100% (13/13)	7.69% (1/13)	–	–	Chemo	2	--	0	--	0
Lei J et al, 2020 ^17^	randomised, controlled, phase II trial	14	7 (50%)	--	IIIA-IIIB	–	28.6% (2/7)	86.7% (6/7)	–	57.1% (4/7)	–	–	camrelizumab+Chemo	3	--	--	--	0
		13	6 (46.2%)			–	16.7% (1/6)	57.1% (4/6)	–	16.7% (1/6)	–	–	Chemo	3	--	--	--	0
Altorki, N. K. et al, 2021 ^18^	randomized, phase II	30	26 (86.7%)	AC(53%),SCC(37%),Sarcomatoid(3%), NOS(7%)	I-IIIA	88% (23/26)	6.7% (2/30)	3% (1/30)	90% (27/30)	0	–	–	durvalumab	2	12 cycles	20%(6/30)	--	1
		30	26 (86.7%)			96% (25/26)	53.3% (16/30)	47% (14/30)	96.7% (29/30)	26.6% (8/30)	–	–	durvalumab+RT	2	12 cycles	23.3%(7/30)	--	1
Qiu FM et al,2022^19^	Randomized, phase II	60	--	NSCLC	IB-IIIA	91.7% (55/60)	41.4% (12/29)	55.2% (16/29)	–	24.1% (7/29)	–	–	Two cycles sintilimab+Chemo		1 y	5%(3/60)	--	--
							26.9% (7/26)	50% (13/26)	–	19.2% (5/26)	–	–	Three cycles sintilimab+Chemo				--	--
Hou X et al,2022 ^20^	Prospective, observational study	31	31 (100.0%)	SCC(48.3%)AC(45.2%)Others(6.5%)	IIIA-IIIB	–	61.3% (19/31)	64.5% (20/31)	100% (31/31)	25.8% (8/31)	95.0% (1-year)	91.6% (1-year)	camrelizumab+ Chemo	3	≥2	--	--	--
		25	24 (96.0%)	SCC(60.0%)AC(40.0%)		–	37.5% (9/24)	40.0% (10/25)	92.0% (23/25)	8.3% (2/24)	83.2% (1-year)	57.0% (1-year)	Chemo	3	≥2	--	--	--
Liang, H et al, 2021 ^21^	retrospective	10	10 (100.0%)	SCC(60%),AC(20%),large-cell(5%),Others(15%)	IIB-IIIB	–	50% (5/10)	80% (8/10)	100% (10/10)	10% (1/10)	100% (10/10)	100% (10/10)	PD-1 inhibitors+Chemo	1-6	--	0	0	0
		10	10 (100.0%)			–	30% (3/10)	30% (3/10)	80% (8/10)	0	60% (6/10)	60% (6/10)	Chemo	1-6	--	--	--	0
Liu Z et al, 2021 ^22^	retrospective	79	79 (100.0%)	SCC(55.9%),AC(32.4%),ASCC(2.9%),large-cell(5.9%),sarcomatoid(1.2%),others (1.8%)	IB-IIIB	100% (79 /79)	53.2% (42/79)	70.9% (56/79)	98.7% (78/79)	–	–	13.28 months (median), 67.2% (2-year)	pembrolizumab/nivolumab/sintilimab/camrelizumab+Chemo	3 (2-5)	--	--	--	--
		91	91 (100.0%)			100% (91 /91)	14.3% (13/91)	47.3% (43/91)	95.7% (87/91)	–	–	12.6 months (median),39.5% (2-year)	Chemo	2 (2-5)	--	--	--	--
Zhao D et al, 2022^23^	retrospective	42	42 (100.0%)	SCC(69.0%)AC(19.0%)Others(11.9%)	IB-IIIB	100% (42/42)	71.4% (30/42)	59.5% (25/42)	80.9% (34/42)	40.5% (17/42)	–	–	pembrolizumab + Chemo	2-4	--	--	--	1
		98	98 (100.0%)	SCC(54.1%)AC(37.8%)Others(8.2%)		100% (98/98)	14.3% (14/98)	22.4% (22/98)	71.4% (70/98)	6.1% (6/98)	–	–	Chemo	2-4	--	--	--	3
Huang Z et al, 2021 ^24^	Retrospective	25	24 (96.0%)	AC(66.3%), SCC(24.8%), Others(8.9%)	IIIA	95.8% (23/24)	37.5% (9/24)	32.0% (8/25)	96.0% (24/25)	4.2% (1/24)	–	–	nivolumab	2	--	12%(3/25)	12.5%(3/24)	0
		82	78 (95.1%)			84.6% (66/78)	12.8% (10/78)	53.7% (44/82)	95.1% (78/82)	2.6% (2/78)	–	–	Chemo	2	--	20.7%(17/82)	16.7%(13/78)	0
Chaft J E et al, 2022^25^	prospective, single-arm	181	159 (88%)	SCC(38%), Others(62%)	IB-IIIB	86.2% (137/159)	20.3% (29/143)	6.1% (11/181)	87.3% (158/181)	5.6% (8/143)	–	–	atezolizumab	1-2	--	11.0%(20/181)	--	--
Zhang Y. et al, 2022^26^	prospective, single-arm	26	17 (65.4%)	SCC	IIB-IIIB	–	38.5%(10/26)	–	–	19.2% (5/26)	–	–	Camrelizumab+Chemo	2-4	--	7.6%(2/26)	--	--
Bahce I. et al, 2022^27^	prospective, single-arm	26	24 (92.3%)	NSCLC	IIB-IIIB	–	79.2% (1924)	–	–	62.5% (15/24)	–	–	Ipilimumab+nivolumab+Chemo+RT	--	--	54%(14/26)		
Gao Y et al, 2022^28^	prospective, single-arm	44	44 (100.0%)	NSCLC	III	100.0% (44/44)	84.1% (37/44)	–	–	59.1% (26/44)	–	–	PD-1 / PD-L1 inhibitors+Chemo	3	--	18.2%(8/44)	--	--
Lin YB et al,2022 ^29^	prospective, single-arm	37	27 (73.0%)	SCC(78.4%) Others(21.6%)	IIB-III	96.0% (26/27)	81.5% (22/27)	–	–	48.1% (13/27)	–	–	tislelizumab+ Chemo	3-4	--	2.7%(1/37)	--	--
Yan S et al,2022 ^30^	prospective, single-arm	53	39 (73.6%)	SCC(79.2%) Others(20.8%)	IIB-IIIB	100.0% (39/39)	61.4% (25/39)	85.7% (42/49)	–	51.3% (20/39)	–	–	tislelizumab+ Chemo	2-4	--	30.6%(15/49)	--	--
Zhang Y et al,2022 ^31^	prospective, single-arm	40	26 (65.0%)	SCC(87.9%) Others(12.1%)	IIIA-IIIB	100.0% (26/26)	57.7% (15/26)	–	–	42.3% (11/26)	–	89.4% (1-year)72.9% (2-year)	toripalimab+ Chemo	2-4	2 cycles+13 cycles	--	--	--
Wang J et al.2021 ^32^	prospective, single-arm	72	72 (100.0%)	SCC(1.4%),AC(6.9%),SCLC(91.7%)	IIIA	–	–	94.4% (68/72)	98.6% (71/72)	29.1% (21/72)	–	–	PD-1 inhibitors+Chemo	2	--	19.4%(14/72)	--	0
Duan H et al.2021 ^33^	prospective, single-arm	23	20 (87.0%)	AC(17.4%), SCC(82.6%)	IIA-IIIB	95% (19/20)	50% (10/20)	73.9% (17/23)	100% (23/23)	30% (6/20)	–	11.3 months (median)	PD-1 inhibitors+Chemo	1-4	--	30%(6/20)	--	--
Zhang, Y. et al, 2021^34^	retrospective	56	45 (80.4%)	NSCLC	IIIA-IIIB	100% (45/45)	68.9% (31/45)	–	–	40% (18/45)	–	–	pembrolizumab/toripalimab+Chemo	--	--	5.4%(3/56)	--	--
Zinner, R. et al, 2020^35^	prospective, single-arm	13	13 (100.0%)	SCC(69%),non-SCC(31%)	-IIIA	–	46% (6/13)	46% (6/13)	–	38% (5/13)	–	–	nivolumab+Chemo	3	--	30.8%(4/13)	--	--
Provencio et al.2020 ^36^	prospective, single-arm, phase II	46	41 (89.1%)	SCC(35%),AC(57%),NOS(9%)	IIIA	100% (41/41)	83% (34/41)	76.1% (35/46)	100% (46/46)	63% (26/41)	97.8% (1-year)93.5% (18 months)89.9% (2-year)	95.7% (1-year)87% (18 months)77.1% (2-year)	nivolumab+Chemo	3	--	30%(14/46)	--	--
Forde PM et al.2018 ^37^	prospective, single-arm	22	21 (95.5%)	AC(62%),SCC(29%),others(10%)	I-IIIA	95% (20/21)	45% (9/20)	9.5% (2/21)	95.2% (20/21)	15% (3/20)	–	RFS:73.0% (18 months)	nivolumab	1	--	4.5%(1/22)	--	--
Bott MJ et al.2018 ^38^	prospective, single-arm, phase I	22	20 (90.9%)	AC(67%),SCC(24%),ASCC(5%), Pleomorphic(5%)	I-IIIA	–	45% (9/20)	9.5% (2/21)	95.2% (20/21)	–	–	–	nivolumab	2	--	5%(1/20)	--	--
Shen D et al, 2021 ^39^	prospective, single-arm	37	37 (100.0%)	SCC(100%)	IIB-IIIB	100% (37/37)	64.9% (24/37)	86.5% (32/37)	100% (37/37)	45.9% (17/37)	–	–	pembrolizumab+Chemo	2	--	--	--	--
Eichhorn F et al, 2021 ^40^	prospective, single-arm, phase II	15	15 (100.0%)	AC(86.7%), SCC(13.3%)	II-IIIA	100% (15/15)	13.3% (2/15)	26.7% (4/15)	93.3% (14/15)	13.3% (2/15)	–	–	pembrolizumab	2	--	20%(3/15)	--	--
Bar, J., et al, 2021^41^	prospective, single-arm	26	23 (88.5%)	AC(50%),SCC(42%),ASCC(4%),NSCLC(4%)	I-II	–	27% (7/26)	4% (1/26)	84.6% (22/26)	12% (3/26)	–	–	pembrolizumab	--	--	8%(2/26)	--	--
Tong BC et al, 2021 ^42^	prospective, single-arm, phase II trial	30	25 (83.3%)	AC(33%),SCC(57%),others(10%)	IB-IIIA	88% (22/25)	28% (7/25)	–	–	12% (3/25)	–	–	pembrolizumab	2	4 cycles	3.3%(1/30)	--	--
Shu CA et al, 2020 ^43^	prospective, single-arm, phase II	30	29 (96.7%)	SCC(40%),AC(57%),Large cell neuroendocrine(3%)	IB-IIIA	87% (26/30)	57% (17/30)	63.3% (19/30)	93.3% (28/30)	33% (10/30)	–	17.9 months (median)	atezolizumab+Chemo	2-4	--	--	--	3
Lee J et al, 2021 ^44^	prospective, single-arm, phase II	181	159 (87.8%)	SCC(38%),NSCC(62%)	IB-IIIB	91% (145/159)	20% (30/147)	–	–	7% (10/147)	–	–	atezolizumab	2	--	9(5.0%)	20(12.6%)	1
Zhang P et al, 2022^45^	prospective, single-arm, phase II trial	50	30 (60.0%)	AC(22%),SCC(56%), NOS(22%)	IIIA	100% (30 /30)	43.3% (13/30)	46% (23/50)	96% (48/50)	20% (6/30)	93.7% (1-year)	85.3% (1-year)	sintilimab+Chemo	2-4	--	8%(4/50)	--	1
Gao S et al, 2020 ^46^	prospective, single-arm, phase Ib	40	37 (92.5%)	SCC(82.5%),AC(15%),Mixed(2.5%)	IA-IIIB	97.3% (36/37)	40.5% (15/37)	20% (8/40)	90% (36/40)	16.2% (6/37)	–	–	sintilimab	2	--	10%(4/40)	--	1
Tao XL et al, 2020 ^47^	prospective, single-arm, phase Ib	36	36 (100.0%)	AC(16.7%),SCC(80.6%),Mixed(2.8%)	IA-IIIB	–	36.1% (13/36)	36.1% (13/36)	94.4% (34/36)	13.9% (5/36)	**–**	**–**	sintilimab	2	**--**	**--**	**--**	**--**
Zhu, X et al, 2021^48^	prospective, single-arm	48	22 (45.8%)	SCC(64.6%), AC(18.8%),NSCLC(16.6%)	IIA-IIIC	100% (22/22)	40.9% (9/22)	62.5% (30/48)	79.2% (38/48)	18.2% (4/22)	**–**	**–**	toripalimab+Chemo	2-4	**--**	6.2%(3/48)	**--**	**--**
Wu YL et al, 2022 ^49^	prospective, single-arm, phase 1b/3	37	34 (91.9%)	AC(13.5%), SCC(83.8%),ASCC(2.7)	II-IIIB	94.1% (32/34)	55.9% (19/34)	70.3% (26/37)	97.3% (36/37)	32.4% (11/34)	–	–	PD-1 inhibitors+Chemo	3	16 cycles	78.4%(29/37)	17.6%(6/34)	
Zhao ZR et al, 2021 ^50^	prospective, single-arm, phase II	33	30 (90.9%)	NSCLC	IIIA-IIIB	96.7% (29/30)	66.7% (20/30)	87.9% (29/33)	97% (32/33)	50% (15/30)	–	–	toripalimab+Chemo	3	12 months	6%(18.2)	--	--
Hong MH et al, 2021 ^51^	prospective, single-arm, phase II	14	11 (78.6%)	AC(50%),others(50%)	III	100% (11/11)	72.7% (8/11)	–	–	27.3% (3/11)	–	–	durvalumab+Chemo+RT	2	12 months	7%(1/14)	--	--
Rothschild SI et al, 2021^52^	prospective, single-arm, phase II trial	67	55 (82.1%)	SCC(33%),AC(55%),large-cell neuroendocrine carcinoma(2%),NOS(10%)	IIIA	93% (51/55)	62% (34 /55)	58% (36 /62)	100% (55/55)	18% (10/55)	91% (1-year)83% (2-year)	EFS: 73% (1-year)	durvalumab+Chemo	3	26 cycles	88%(59 /67)	87%(48 /55)	2
Tfayli A et al, 2020 ^53^	prospective, single-arm, phase II	15	11 (73.3%)	SCC(13.3%), AC(86.7%)	IB-IIIA	–	9.1% (1/11)	27.2% (3/11)	81.8% (9/11)	6.7% (1/15)	–	–	avelumab+Chemo	3	--	26.7%(4/15)	--	--
Deng H et al, 2021 ^54^	retrospective	51	31 (60.8%)	SCC(77.4%), AC(19.4%), ASCC(3.2%), large cell lung cancer(3.2%), lymphoepithelioma-like carcinoma(6.5%)	IIIB	96.8% (30 /31)	67.8% (21/31)	71% (22/31)	100% (31/31)	35.5% (11/31)	–	27.5 months (median)	sintilimab/pembrolizumab/camrelizumab/nivolumab/tislelizumab+Chemo	3.4	--	--	--	--
Shi, L., et al, 2021^55^	retrospective	27	27 (100.0%)	SCC	IIA-IIIB	–	55.6% (15/27)	–	–	29.6% (8/27)	–	–	sintilimab,/pembrolizum/camrelizumab/toripalimab/tislelizumab+Chemo	1-4	--	66.6%(18/27)	--	--
Hong T et al, 2021 ^56^	retrospective	25	25 (100.0%)	SCC(76%), AC(20%), Others(4%)	IIA-IIIC	100% (25/25)	52% (13/25)	88% (22/25)	100% (25/25)	32% (8/25)	–	–	sintilimab/pembrolizumab/camrelizumab+Chemo	3	--	--	--	--
Hu Y et al, 2021^57^	retrospective	20	20 (100.0%)	AC(20%), SCC(70%), ASCC(5%), large-cell neuroendocrine carcinoma(5%)	IB-IIIB	100% (20/20)	40% (8/20)	75% (15/20)	100% (20/20)	25% (5/20)	–	–	sintilimab/pembrolizumab/tislelizumab/toripalimab++Chemo	2-4	--	0	--	--
Cheng X. et al, 2022^58^	retrospective	19	19 (100%)	SCC(63.2%),Other(36.8%)	IIIA-IIIB	100% (19/19)	79.0% (15/19)	–	–	52.6% (10/19)	–	–	Tislelizumab+Chemo	2-4	--	--	--	--
Chen T et al, 2021 ^59^	retrospective	12	12 (100.0%)	SCC(33.3%), AC(50.0%), ASCC(8.3%)Undifferentiated adenocarcinoma (8.3%)	IIIA-IIIB	100% (12/12)	33.3% (4/12)	50% (6/12)	100% (12/12)	41.7% (5/12)	–	–	pembrolizumab/nivolumab+Chemo	--		--	--	--
Wu J et al, 2022^60^	retrospective	76	76 (100.0%)	AC(22%), SCC(65%), NSCLC-NOS(13%)	IB-IIIB	100% (76/76)	64% (49/76)	75% (57/76)	100% (76/76)	37% (28/76)	–	–	pembrolizumab/nivolumab+Chemo	2-4	--	25%(19 /76)	--	--
Zhang, Y et al, 2021^61^	retrospective	30	23 (76.7%)	SCC(73.3%), others(26.7%)	IIIA-IIIB	100% (23/23)	69.6% (16/23)	–	–	30.4% (7/23)	–	–	pembrolizumab/toripalimab+Chemo	2	--	10%(3/30)	--	--
Chen Y et al, 2021 ^62^	Retrospective, single-arm	35	35 (100.0%)	SCC(74.3%), AC(20%), Large-cell carcinoma(2.9%),Sarcomatoid carcinoma(2.9%)	IIIA-IIIB	100% (35/35)	74.3% (26/35)	48.6% (17/35)	100% (35/35)	51.4% (18/35)	–	–	pembrolizumab+Chemo	2	--	2.9%(1/35)	--	--
Zhai H et al, 2022 ^63^	retrospective	46	45 (97.8%)	AC(41.3%), SCC(58.7%)	IIIA-IIIB	95.6% (43/45)	17.8% (8/45)	60.9% (28/46)	97.8% (45/46)	53.3% (24 /45)	90.5% (1-year), 86.8% (18 months)79.9% (2-year)	67% (1-year), 53.4% (18 montyhs)45.8% (2-year)	nivolumab+Chemo	3	at least 1 cycle	19.6%(9/46)	--	--
Yao Y et al, 2022 ^64^	retrospective	11	11 (100%)	AC(8.9%), SCC(91.1%)	IIIA-IIIB	100% (11/11)	81.8% (9/11)	72.7% (8/11)	100% (11/11)	72.7% (8/11)	–	–	camrelizumab/ durvalumab+Chemo	2-3	1-2 cycles	--	--	--
Fan BS et al,2022 ^65^	retrospective	8	8 (100.0%)	SCC(75.0%)AC(25.0%)	IIIA/IIIB	100.0% (8/8)	62.5% (5/8)	87.5% (7/8)	100% (8/8)	–	–	–	sintilimab+Chemo	1-3	--	12.5%(1/8)	--	--

Therefore they were included in our meta-analysis due to different purposes.

AC, adenocarcinoma; AEs, adverse events; ASCC, adenocarcinoma squamous cell cancer, Chemo:Chemotherapy; DCR, disease control rate; DFS, disease-free survival; EFS, event-free survival; G3, grade 3; MPR, major pathological response; NOS, Not otherwise specified; NR, not reached; NSCLC, non small cell lung cancer; ORR, objective response rate; OS, overall survival; pCR, pathological complete response; PFS, progression-free survival; R0, R0 resection (no residual tumor); RFS, recurrence-free survival; RT, Radiotherapy; SCC, squamous cell cancer; SCLC, small cell lung cancer; SRAE, surgery-related adverse events; TRAE, treatment-related adverse events.

### MPR of neoadjuvant immunotherapy

**Figure 2:**
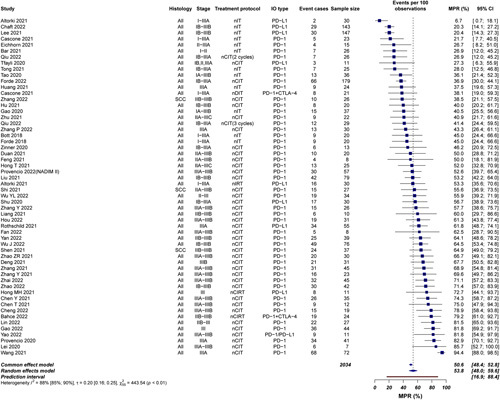
Weighted random-effects model of major pathological response (MPR). Of note, Cascone *et al*.^14^ and Altorki *et al*.^18^ reported MPR rates of neoadjuvant ICI alone and ICI combination regimens, and Qiu ^19^ reported MPR rates of neoadjuvant ICI 2 cycles and 3 cycles; therefore, for all these studies, data of each were analyzed as two groups.

Fifty-three articles were included. Of note, Cascone *et al*.^14^ and Altorki *et al*.^18^ reported MPR rates of neoadjuvant ICI alone and ICI combination regimens, and Qiu^19^ reported MPR rates of neoadjuvant ICI 2 cycles and 3 cycles. For these three articles, each arm needed to be analyzed as an independent data. After pooled analysis of the 56 MPR data, the estimated rate was 53.8% (95% CI 47.0–59.6%, 95% PI 16.9–88.4%; *I* ^2^=88% 95% CI 85–90%; τ=0.20, 95% CI 0.16–0.25) (Fig. 2).

### Comparison MPR of nCIT vs nCT alone

There were eight comparative studies from the available data; of these, the most common comparison was nCIT versus nCT alone (*n*=8; five prospective and three retrospective).

**Figure 3:**
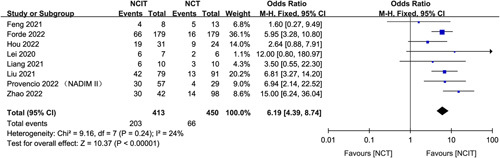
Pooled analyses of MPR comparing between nCIT and standard nCT.

As compared to nCT, patients who underwent nCIT were associated with higher MPR rates; the OR was 6.19 (95% CI 4.39–8.74, *P*<0.00001) (Fig. 3). There was no significant heterogeneity.

### Correlation between MPR and survival outcome

**Figure 4:**
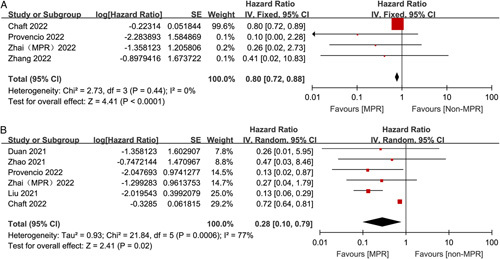
Association between MPR and time-to-event outcomes.

We explored whether MPR could be an indicator for survival. As a result, HR for DFS/PFS/EFS and OS by MPR status crossed six studies and four studies, respectively. The overall HR for DFS/PFS/EFS was 0.28 (95% CI: 0.10–0.79, *P*=0.02), indicating a statistically significant association between MPR and DFS/PFS/EFS (Fig. 4). The overall HR for OS was 0.80 (95% CI: 0.72–0.88, *P*<0.0001), indicating a statistically significant association between MPR and OS (Fig. 4).

### Correlation between MPR and ORR

**Figure 5:**
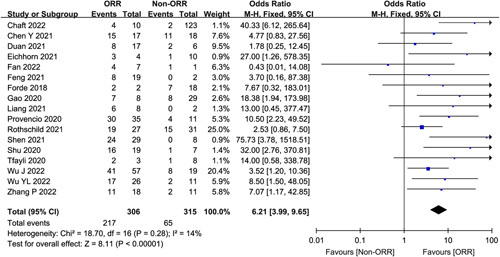
Association between MPR and ORR.

Although a minority of studies reported the relationship between MPR and ORR (*n*=17), the results were statistically significant and all without heterogeneity. MPR was associated with a OR of 6.21 for ORR (95% CI 3.71–9.16, *P*<0.00001) (Fig. 5).

### Predictors of MPR and association with outcomes

**Figure 6:**
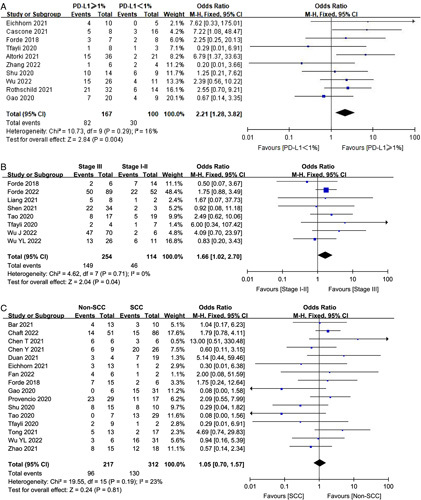
Major pathological response rates by PD-L1 expression, clinical stage and histology.

Lastly, we examined several potential predictors of developing MPR with neoadjuvant immunotherapy (with or without other neoadjuvant therapies), such as PD-L1 expression, histology, and clinical stage. Ten studies reported MPR rates by PD-L1 tumor proportion score. Patients with PD-L1 ≥1% were significantly more likely to achieve MPR (OR 2.21, 95% CI 1.28–3.82, *P*=0.004, Fig. 6) than that with PD-L1 negative (<1%). Eight studies reported MPR rates by clinical stage (III vs I/II). Patients with stage III were significantly more likely to achieve MPR (OR 1.66, 95% CI 1.02–2.70, *P*=0.04, Fig. 6) than stage I/II patients. Histology (squamous cell vs nonsquamous) was not significantly associated with MPR rates (Fig. 6). There was no heterogeneity in any of the aforementioned parameter.

### Sensitivity analysis and statistical analysis of publication bias

Sensitivity analysis by systematically eliminating each specific study from the total count demonstrated that the new HRs or ORs were similar to the original HRs/ORs as above (Supplemental Table 7, Supplemental Digital Content 10, http://links.lww.com/JS9/A624), implying that any given study might not have disproportionately impacted the results. Additionally, statistical analysis of publication bias demonstrated no statistically significant evidence thereof (Supplemental Figure S3, Supplemental Digital Content 11, http://links.lww.com/JS9/A625).

## Discussion

Neoadjuvant therapy has the potential to improve the survival of resectable NSCLC patients. The challenge remains to determine the best neoadjuvant approach to achieve a high response rate and acceptable toxicity. Immunotherapy recently emerges as a promising therapeutic strategy for NSCLC, and some focus has shifted to the use of ICI in early-stage NSCLC. The delivery of early ICI therapy may lead to a deep pathological response because of the antigen load of the entire cancer prior to surgical resection^66,67^. Brandt *et al*.^68^ reported that the MPR rate following nCT was 15%. The CheckMate 159 research showed that neoadjuvant nivolumab achieved MPR in 45% of participants^37^. The phase II NADIM study evaluated the effectiveness of nivolumab combined with carboplatin/paclitaxel as neoadjuvant therapy in patients with stage IIIa resectable NSCLC. A high MPR rate of 82.9% suggested that nCIT might be a new option for patients with locally advanced NSCLC^36^. It was determined that nivolumab plus chemotherapy showed statistically significant improvement compared to neoadjuvant platinum-based chemotherapy alone in the MPR as a neoadjuvant treatment for resectable NSCLC in the phase III CheckMate-816 trial by 36.9 versus 8.9%, respectively^9^. In our meta-analysis, the pooled MPR was 53.8%, which demonstrated the addition of immunotherapy to nCT could improve pathological responses obviously. Our data confirmed that nCIT yielded promising efficacy, with an increased MPR rate compared with chemotherapy alone (49.2 vs 14.7%). The synergistic effect of chemotherapy and ICIs, with the cytotoxic chemotherapy increasing the recognition of these agents as immunotherapies, might explain the high rates of MPR^69,70^.

The median time from trial initiation to publication for adjuvant platinum chemotherapy trials in NSCLC was longer than 10 years^11^. Early and widespread acceptance of new perioperative treatment strategies for resectable NSCLC was usually hindered by a lack of surrogate endpoints of clinical efficacy. Pathological response has shown patient-level association with survival in various cancers^71–73^, which requires further evaluation across ongoing trials of neoadjuvant therapy involving patients with NSCLC.A meta-analysis based on 21 clinical studies showed that the HR of OS under different pCR states was 0.49 (95% CI 0.43–0.56)^74^. Another combined analysis of two nCT studies showed that, 5-year OS was 80.0% in the pCR group versus 55.8% in the non-pCR group (*P*=0.0007), and pCR was a favorable prognostic factor of OS (HR 0.34; 95% CI 0.18–0.64)^72^. Maybe the low pCR rate after neoadjuvant treatments and insufficient data were available for analysis, its use was greatly limited as a surrogate endpoint. Compared with pCR, MPR is seemed more common. Although without mediastinal downstaging evaluation, MPR has been accepted as another surrogate of survival in patients with NSCLC received nCT. William *et al*.^74^ reported the MPR rate following nCT was 30% and histopathologic response was a significant predictors of OS. The College of American Pathologists recommended MPR as one of the study endpoint of clinical trials on neoadjuvant immunotherapy for lung cancer. However, the relationship between OS and MPR in resectable NSCLC patients who receiving neoadjuvant immunotherapy has not been fully elucidated. The present meta-analysis synthesized the results of published clinical trials, we found that MPR were shown to be associated with improved OS (HR=0.80, 95% CI: 0.72–0.88, *P*<0.0001) and DFS/PFS/EFS (HR=0.28, 95% CI: 0.10–0.79, *P*=0.02) when compared with non-MPR. MPR seemed to be an alternative endpoint of OS in patients with NSCLC received nCT.

In the MRC LU22/NVALT 2/EORTC 08012 multicenter randomised trial, nCT resulted in a good radiological response rate (4% CR, 45% PR). However, there was no evidence of a benefit in terms of OS. The discrepancy between the radiographic and pathological assessment was often observed. The tumor response patterns of Immune agents may differ compared with conventional chemotherapeutic agents^75^. The incidence of radiographic partial response and complete response with nCIT ranged from 38 to 72%^7,36,43,52,77^. Pseudo progression, which was characterized by radiologic progression of the tumor burden, followed by objective response, was first described in melanoma patients treated with ipilimumab^78^. Some studies indicated that pseudo progression may occur in other cancer types receiving ICI therapy. This unconventional phenomenon does not typically occur with traditional cytotoxic chemotherapy. In the present study, objective response with neoadjuvant immonotherapy indicated an increased likelihood of MPR (OR=6.21, 95% CI 3.71–9.16, *P*<0.00001). Although new immunotherapy-specifc radiologic criteria has been developed, the classic RECIST remains a reasonable and meaningful method to assess response to immunotherapy in the clinic^79^.

Recent trials had evaluated potential predictive biomarkers for MPR, but there was no consensus currently. Two dominant subtypes, accounting for ~80% of NSCLC cases, are lung adenocarcinoma and lung squamous carcinoma (SCC)^80^. Several studies have reported relatively higher MPR rates in SCC patients, compared with adenocarcinoma^43,81^. In our pooled analysis, there was no difference (44.2 vs 41.7%, *P*=0.81) in MRP rates between SCC and non-SCC.

Usually, patient with earlier-stage disease (stages IB to II) are recommended for upfront resection and adjuvant chemotherapy based on a series of large prospective trials and the Lung Adjuvant Cisplatin Evaluation meta-analysis^82^. It is still unclear as to which stages of NSCLC benefit the most from neoadjuvant ICI therapy. A stage-based assessment of pathological responses is important as it may allow for improved design of future trials in specific disease stages^69^. As reported in the CheckMate-816 trial (NCT02998528), the magnitude of EFS benefit was greater for the patients with stage IIIA (HR 0.54) than for those with stages IB to II disease (HR 0.87) and for patients with tumor PD-L1 expression of 1% or greater (HR 0.41) than for those with PD-L1 expression lower than 1% (HR 0.85). The MPR benefit for stage IIIA patients with the addition of nivolumab in CheckMate-816 was more impressive than that for stage IB to stage II^9^. In our meta-analysis, eight studies explored MPR rates of different stages, and similar to the previous perioperative chemotherapy, patients with stage III were significantly more likely to achieve MPR (OR=1.66, 95% CI 1.02–2.70, *P*=0.04).

Irrespective of the results in metastatic stage IV patients, the predictive value of the PD-L1 status might have a different impact in patients with non-metastatic earlier-stage lung cancer with less tumor burden^40^. Both the NEOSTAR^14^ and the Checkmate-816 trial^9^ showed that elevated PD-L1 expression was also associated with higher pathologic responses. However, both the CLMC3 trial^37^ and Shu *et al*.^43^. found no association between pathological response and PD-L1 expression. In our study, patients with stage III (vs I/II) or PD-L1 ≥1% (vs PD-L1<1%) were significantly more likely to achieve MPR, and histology (SCC vs non-SCC) was not significantly associated with MPR.

There were certain limitations to this meta-analysis. First of all, no prospective study data directly confirmed the correlation between pathological response and survival of neoadjuvant immunotherapy, most of the included trials were nonrandomized single-arm clinical trials with a small sample size, and these corresponding analyses were based on indirect comparisons. Second, so far, there was still no standardized method for MPR evaluation, especially when immunotherapy was added into neoadjuvant therapy. An ideal observation index should be easy to measure, and with a smaller chance of having a deviation, whereas neoadjuvant immunotherapy was different from the other treatments with more diverse pathological changes. Third, in several studies, the criteria of MPR were not quite uniform. In Checkmate-816 trial, the evaluation of MPR included sampled lymph nodes. Fourth, MPR might be differ after neoadjuvant immunotherapy between squamous cell carcinoma and adenocaicinoma, although there was no difference in our result, the optimal cutoff value for pathological response might require further refinement. Additionally, optimized methods of histologic sampling, interobserver variability in the assessment of pathological response, varied cycles of neoadjuvant therapy would affect the final pathological results. What’s more, some of the included trials are still ongoing with only initial results, more prospective studies are needed to confirm its validity and its relationship with DFS and OS. Despite the above deficiencies, to our knowledge, this is the first meta-analysis to evaluate MPR as a surrogate marker of improved long-term outcomes of NSCLC receiving immunotherapy. Once a recognized surrogate endpoint is established, it would accelerate clinical trials and drug development.

## Conclusions

Results of this systematic review and meta-analysis demonstrate that nCIT achieved higher MPR in NSCLC patients, and increased MPR might be associated with survival benefits. It seems that the MPR could serve as a surrogate endpoint of survival to evaluate neoadjuvant immunotherapy. In the future, more randomized clinical trials are warranted to confirm our conclusions.

## Ethical approval

No.

## Conflicts of interest disclosure

The authors have no conflicts of interest.

## Sources of funding

No.

## Author contribution

Y.C. and J.Q.: methodology, software, validation, investigation, resources, data curation, writing-original draft; Y.W.: methodology, software, investigation, data curation; Q.L.: conceptualization, investigation, resources, data curation, writing-review and editing; J.W. and W.Z.: conceptualization, investigation, data curation; F.L.: conceptualization, methodology, software and validation; Z.H.: conceptualization, data curation, investigation; M.Z.: conceptualization, data curation, investigation; J.W.: conceptualization, methodology, validation, investigation, data curation, writing-review and editing, visualization, supervision, project administration, funding acquisition.

## Guarantor

Jun Wang.

## Supplementary Material

SUPPLEMENTARY MATERIAL
