# Supplementary material for: Does major pathological response after neoadjuvant Immunotherapy in resectable nonsmall-cell lung cancers predict prognosis? A systematic review and meta-analysis
Source: Int J Surg. 2023 May 26;109(9):2794–807. doi: 10.1097/JS9.0000000000000496 (PMC10498860; doi:10.1097/JS9.0000000000000496)
Supplement: SUPPLEMENTARY MATERIAL [file js9-109-2794-s002.docx]

**Table S7 Sensitivity analysis for MPR when comparing neo-chemoimmunotherapy with neo-chemotherapy and RFS & OS& ORR by MPR or not**

| Items | Study omitted | Estimate | 95%CI | |
| --- | --- | --- | --- | --- |
| MPR | Forde et al | 6.33 | 4.15 | 9.64 |
|  | Lei et al | 6.13 | 4.33 | 8.67 |
|  | Feng et al | 6.52 | 4.58 | 9.29 |
|  | Liang et al | 6.31 | 4.44 | 8.96 |
|  | Provencio et al | 6.12 | 4.27 | 8.77 |
|  | Hou et al | 6.77 | 4.70 | 9.75 |
|  | Zhao et al  Liu et al | 5.36  6.03 | 3.69  4.09 | 7.80  8.91 |
|  | Combined | 6.19 | 4.39 | 8.74 |
| DFS/PFS/EFS/RFS | Duan et al | 0.28 | 0.09 | 0.85 |
|  | Zhao et al | 0.27 | 0.09 | 0.82 |
|  | Liu et al | 0.56 | 0.32 | 0.97 |
|  | Provencio et al | 0.32 | 0.11 | 0.98 |
|  | Zhai et al | 0.28 | 0.09 | 0.90 |
|  | Chaft et al | 0.16 | 0.08 | 0.30 |
|  | Combined | 0.28 | 0.10 | 0.79 |
| OS | Chaft et al | 0.22 | 0.04 | 1.14 |
|  | Zhai et al | 0.80 | 0.72 | 0.88 |
|  | Zhang et al | 0.80 | 0.72 | 0.88 |
|  | Provencio et al | 0.80 | 0.72 | 0.88 |
|  | Combined | 0.80 | 0.72 | 0.88 |
| ORR | Chaft et al | 5.83 | 3.71 | 9.16 |
|  | Chen Y 2021 | 6.32 | 4.01 | 9.98 |
|  | Duan 2021 | 6.67 | 4.23 | 10.51 |
|  | Eichhorn 2021 | 6.02 | 3.86 | 9.41 |
|  | Fan 2022 | 6.60 | 4.21 | 10.34 |
|  | Feng 2021 | 6.28 | 4.03 | 9.81 |
|  | Forde 2018 | 6.17 | 3.95 | 9.64 |
|  | Gao 2020 | 5.88 | 3.74 | 9.23 |
|  | Liang 2021 | 6.12 | 3.92 | 9.55 |
|  | Provencio 2020 | 5.97 | 3.77 | 9.44 |
|  | Rothschild 2021 | 7.43 | 4.55 | 12.13 |
|  | Shen 2021 | 5.61 | 3.57 | 8.82 |
|  | Shu 2020 | 5.84 | 3.72 | 9.16 |
|  | Tfayli 2020 | 6.12 | 3.92 | 9.55 |
|  | Wu J 2022 | 6.89 | 4.23 | 11.23 |
|  | Wu YL 2022 | 6.06 | 3.84 | 9.57 |
|  | Zhang P 2022 | 6.15 | 3.90 | 9.70 |
|  | Combined | 6.21 | 3.99 | 9.65 |
