# Supplementary figures and images for: Does major pathological response after neoadjuvant Immunotherapy in resectable nonsmall-cell lung cancers predict prognosis? A systematic review and meta-analysis
Source: Int J Surg. 2023 May 26;109(9):2794–807. doi: 10.1097/JS9.0000000000000496 (PMC10498860; doi:10.1097/JS9.0000000000000496)

A

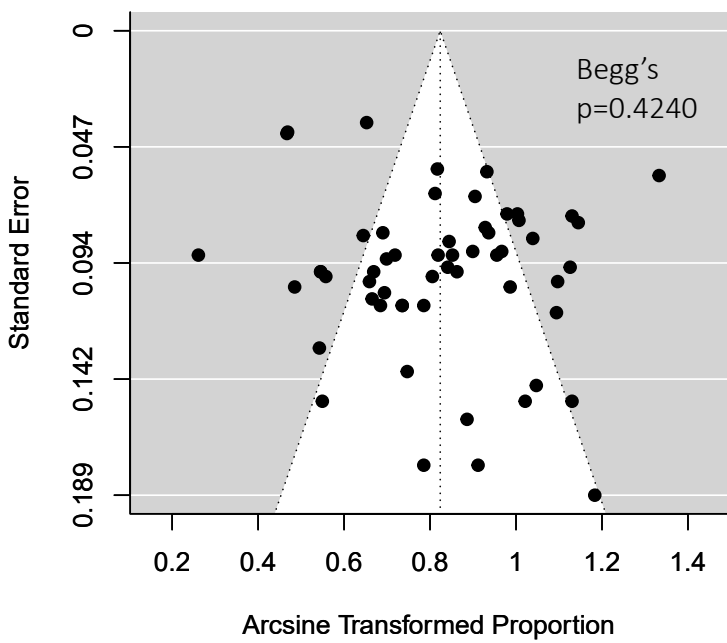

B

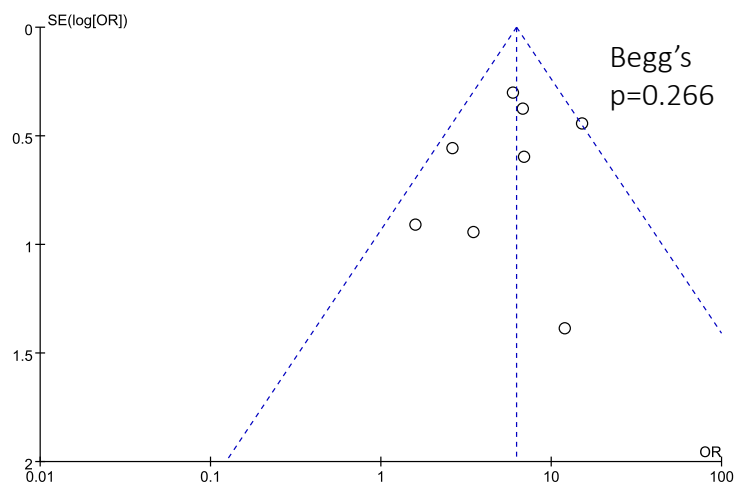

C

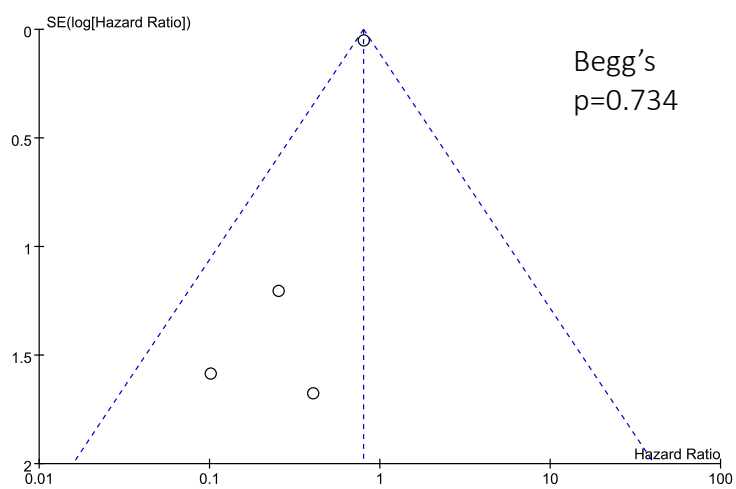

D

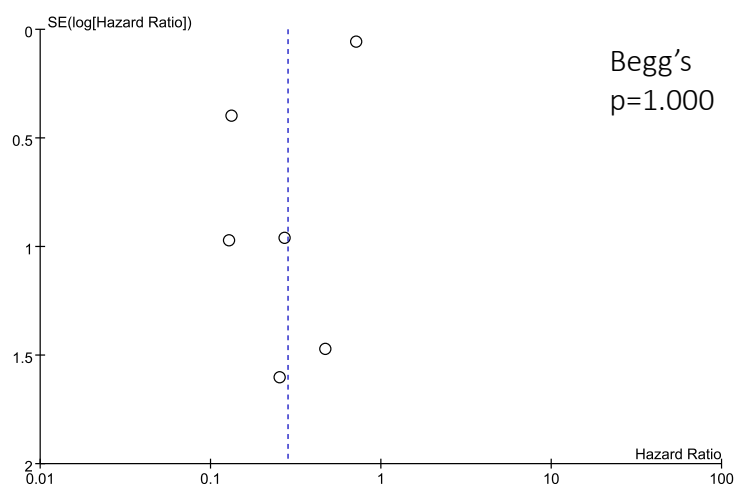

E

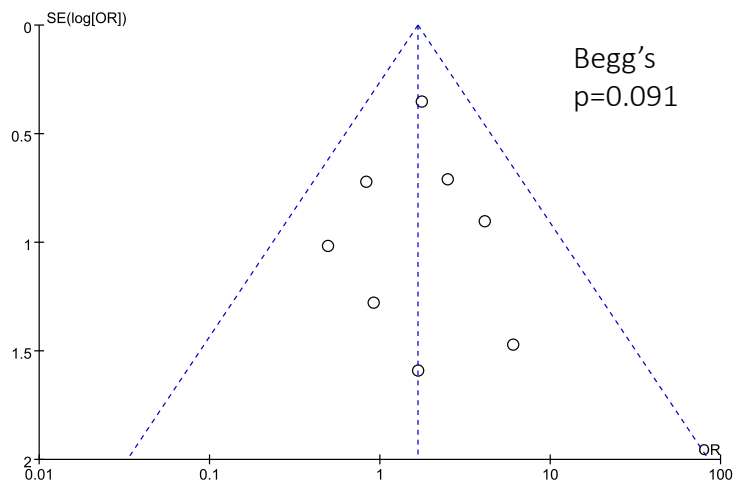

F

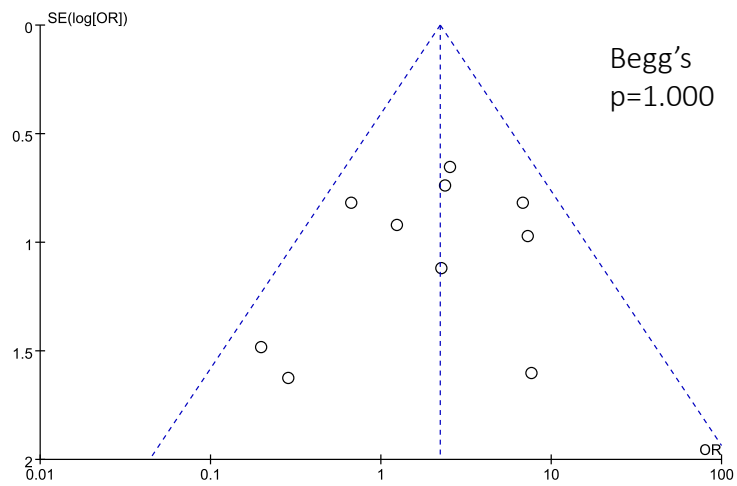

G

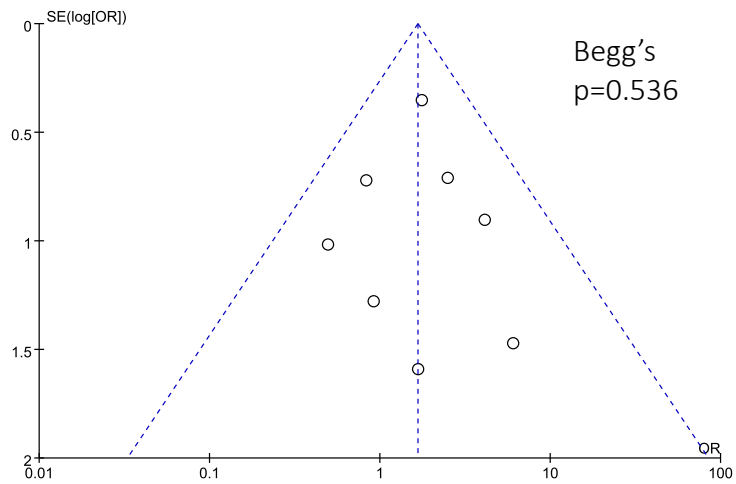

H

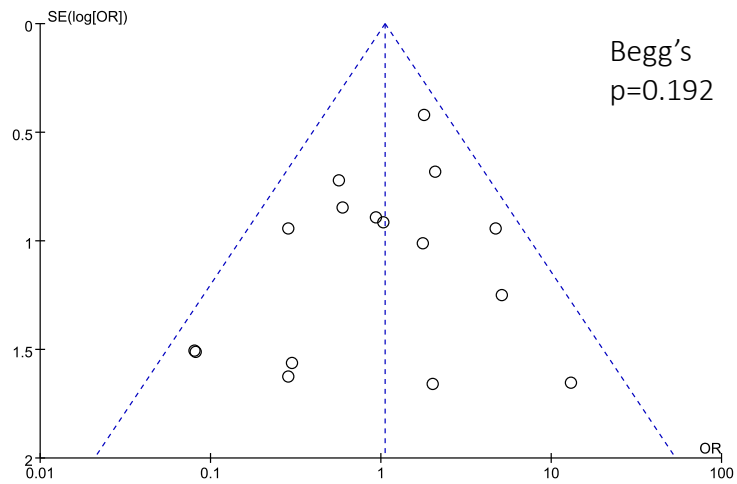

Supplement: SUPPLEMENTARY MATERIAL [file js9-109-2794-s003.pdf]

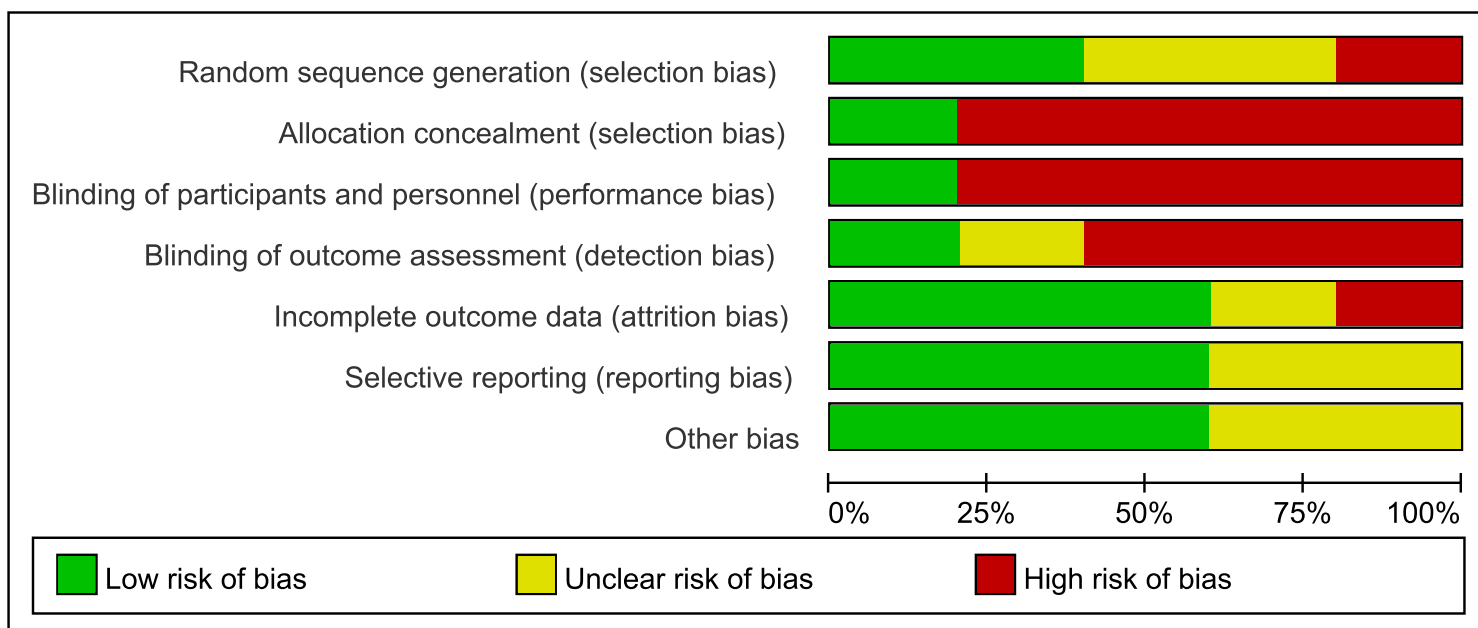

Supplement: SUPPLEMENTARY MATERIAL [file js9-109-2794-s008.pdf]
