# Supplementary material for: Does major pathological response after neoadjuvant Immunotherapy in resectable nonsmall-cell lung cancers predict prognosis? A systematic review and meta-analysis
Source: Int J Surg. 2023 May 26;109(9):2794–807. doi: 10.1097/JS9.0000000000000496 (PMC10498860; doi:10.1097/JS9.0000000000000496)
Supplement: SUPPLEMENTARY MATERIAL [file js9-109-2794-s006.docx]

**Supplementary**

**Table 1. MEDLINE (PubMed) search**

| **Search** | **Query** | **Items found** |
| --- | --- | --- |
| #4 | #1 AND #2 AND #3 | 508 |
| #3 | "Neoadjuvant Therapy"[MeSH] OR neoadjuvant[tiab] OR "neo-adjuvant"[tiab] OR perioperative[tiab] OR preoperative[tiab] OR "peri-operative"[tiab] OR "pre-operative"[tiab] | 458.177 |
| #2 | Immunotherapy[MeSH] OR "Antibodies, Monoclonal, Humanized"[MeSH] OR "Programmed Cell Death 1 Receptor"[MeSH] OR "CTLA-4 Antigen"[MeSH] OR "B7-H1 Antigen"[MeSH] OR "Immune Checkpoint Inhibitors"[MeSH] OR immunotherap*[tiab] OR immuno-therap*[tiab] OR immunetherap*[tiab] OR immune-therap*[tiab] OR immune checkpoint inhibit*[tiab] OR nivolumab[tiab] OR ipilimumab[tiab] OR sintilimab[tiab] OR durvalumab[tiab] OR atezolizumab[tiab] OR pembrolizumab[tiab] OR avelumab[tiab] OR tremelimumab[tiab] OR camrelizumab[tiab] OR tislelizumab[tiab] OR toripalimab[tiab] | 487.643 |
| #1 | "Carcinoma, Non-Small-Cell Lung"[MeSH] OR non-small cell lung cancer*[tiab] OR non-small cell lung carcinoma*[tiab] OR nonsmall cell lung cancer*[tiab] OR nonsmall cell lung carcinoma*[tiab] OR NSCLC[tiab] | 95.110 |
| **Abbreviations:** [MeSH] = Medical Subject Headings; [tiab] = Title/Abstract | |  |

**Table 2. EMBASE search**

| **Search** | **Query** | **Items found** |
| --- | --- | --- |
| #4 | #1 AND #2 AND #3 | 1529 |
| #3 | 'neoadjuvant therapy'/exp OR ‘neoadjuvant’:ab,ti,kw OR 'neo-adjuvant':ab,ti,kw OR ‘perioperative’:ab,ti,kw OR ‘preoperative’:ab,ti,kw OR 'peri-operative':ab,ti,kw OR 'pre-operative':ab,ti,kw | 669112 |
| #2 | 'immunotherapy'/exp OR 'monoclonal antibody'/exp OR 'immune checkpoint inhibitor'/exp OR 'programmed death 1 receptor'/exp OR 'programmed death 1 ligand 1'/exp OR 'cytotoxic t lymphocyte antigen 4'/exp OR ‘immunotherap*’:ab,ti,kw OR 'immuno-therap*':ab,ti,kw OR ‘immunetherap*’:ab,ti,kw OR 'immune-therap*':ab,ti,kw OR 'immune checkpoint inhibit*':ab,ti,kw OR ‘nivolumab’:ab,ti,kw OR ‘ipilimumab’:ab,ti,kw OR ‘sintilimab’:ab,ti,kw OR ‘durvalumab’:ab,ti,kw OR ‘atezolizumab’:ab,ti,kw OR ‘pembrolizumab’:ab,ti,kw OR ‘avelumab’:ab,ti,kw OR ‘tremelimumab’:ab,ti,kw OR ‘camrelizumab’:ab,ti,kw OR ‘tislelizumab’:ab,ti,kw OR ‘toripalimab’:ab,ti,kw | 1007289 |
| #1 | 'non small cell lung cancer'/exp OR 'non-small cell lung cancer*':ab,ti,kw OR 'non-small cell lung carcinoma*':ab,ti,kw OR 'nonsmall cell lung cancer*':ab,ti,kw OR 'nonsmall cell lung carcinoma*':ab,ti,kw OR ‘NSCLC’:ab,ti,kw | 214422 |

**Abbreviations:** /exp = explosion in Emtree terms; :ti,ab,kw = title, abstract, keywords

**Table 3. CENTRAL(Cochrane) research**

| **Search** | **Query** | **Items found** |
| --- | --- | --- |
| #4 | #1 AND #2 AND #3 | 156 |
| #3 | "Neoadjuvant Therapy"[MeSH] OR neoadjuvant[tiab] OR "neo-adjuvant"[tiab] OR perioperative[tiab] OR preoperative[tiab] OR "peri-operative"[tiab] OR "pre-operative"[tiab] | 73973 |
| #2 | Immunotherapy[MeSH] OR "Antibodies, Monoclonal, Humanized"[MeSH] OR "Programmed Cell Death 1 Receptor"[MeSH] OR "CTLA-4 Antigen"[MeSH] OR "B7-H1 Antigen"[MeSH] OR "Immune Checkpoint Inhibitors"[MeSH] OR immunotherap*[tiab] OR immuno-therap*[tiab] OR immunetherap*[tiab] OR immune-therap*[tiab] OR immune checkpoint inhibit*[tiab] OR nivolumab[tiab] OR ipilimumab[tiab] OR sintilimab[tiab] OR durvalumab[tiab] OR atezolizumab[tiab] OR pembrolizumab[tiab] OR avelumab[tiab] OR tremelimumab[tiab] OR camrelizumab[tiab] OR tislelizumab[tiab] OR toripalimab[tiab] | 26298 |
| #1 | "Carcinoma, Non-Small-Cell Lung"[MeSH] OR non-small cell lung cancer*[tiab] OR non-small cell lung carcinoma*[tiab] OR nonsmall cell lung cancer*[tiab] OR nonsmall cell lung carcinoma*[tiab] OR NSCLC[tiab] | 15617 |
| **Abbreviations:** [MeSH] = Medical Subject Headings; [tiab] = Title/Abstract | |  |
