# Supplementary material for: Does major pathological response after neoadjuvant Immunotherapy in resectable nonsmall-cell lung cancers predict prognosis? A systematic review and meta-analysis
Source: Int J Surg. 2023 May 26;109(9):2794–807. doi: 10.1097/JS9.0000000000000496 (PMC10498860; doi:10.1097/JS9.0000000000000496)
Supplement: SUPPLEMENTARY MATERIAL [file js9-109-2794-s010.doc]

**Table 6 The quality assessment of non-RCTs using MINORS score**

| Study | A clearly stated aim | Inclusion of consecutive patients | Prospective collection of data | Endpoints appropriate to the aim of the study | Unbiased assessment of the study endpoint | Follow-up period appropriate to the aim of the study | Loss to follow up less than 5% | Prospective calculation of the study size | Additional criteria in the case of comparative study | | | |
| --- | --- | --- | --- | --- | --- | --- | --- | --- | --- | --- | --- | --- |
|  |  |  |  |  |  |  |  |  | An adequate control group | Contemporary groups | Baseline equivalence of groups | Adequate statistical analyses |
| Feng, Y et al, 2021^[16]^ | 2 | 0 | 2 | 2 | 0 | 2 | 2 | 0 | 0 | 2 | 2 | 2 |
| Lei J et al, 2020 ^[17]^ | 2 | 2 | 2 | 2 | 2 | 1 | 2 | 2 | 2 | 2 | 2 | 2 |
| Liang, H et al, 2021 ^[21]^ | 2 | 1 | 2 | 2 | 0 | 2 | 2 | 0 | 0 | 2 | 2 | 2 |
| Liu Z et al, 2021 ^[22]^ | 2 | 2 | 0 | 2 | 2 | 2 | 1 | 0 | 2 | 2 | 2 | 2 |
| Zhao D et al, 2022 ^[23]^ | 2 | 2 | 2 | 2 | 2 | 2 | 2 | 1 | 2 | 2 | 2 | 2 |
| Huang Z et al, 2021 ^[24]^ | 2 | 2 | 0 | 2 | 2 | 2 | 2 | 0 | 2 | 2 | 2 | 2 |
| Chaft JE et al, 2022^[25]^ | 2 | 2 | 2 | 2 | 2 | 2 | 2 | 2 | -- | -- | -- | -- |
| Zhang Y. et al, 2022^[26]^ | 2 | 2 | 1 | 2 | 2 | 2 | 2 | 2 | -- | -- | -- | -- |
| Bahce I. et al, 2022^[27]^ | 2 | 2 | 1 | 2 | 2 | 0 | 1 | 0 | -- | -- | -- | -- |
| Gao Y et al, 2022 ^[28]^ | 2 | 2 | 2 | 2 | 2 | 1 | 1 | 0 | -- | -- | -- | -- |
| Lin YB et al,2022 ^[29]^ | 2 | 2 | 2 | 2 | 2 | 2 | 2 | 1 | -- | -- | -- | -- |
| Yan S et al,2022 ^[30]^ | 2 | 2 | 2 | 2 | 2 | 1 | 2 | 0 | -- | -- | -- | -- |
| Zhang Y et al,2022 ^[31]^ | 2 | 2 | 2 | 2 | 2 | 2 | 2 | 1 | -- | -- | -- | -- |
| Wang J et al.2021 ^[32]^ | 2 | 2 | 2 | 2 | 2 | 1 | 1 | 0 | -- | -- | -- | -- |
| Duan H et al.2021 ^[33]^ | 2 | 2 | 2 | 2 | 2 | 1 | 1 | 0 | -- | -- | -- | -- |
| Zhang, Y. et al, 2021^[34]^ | 2 | 0 | 1 | 2 | 0 | 2 | 0 | 0 | -- | -- | -- | -- |
| Zinner, R. et al, 2020^[35]^ | 2 | 0 | 0 | 2 | 0 | 1 | 2 | 1 | -- | -- | -- | -- |
| Provencio et al.2020,2022 ^[36,15]^ | 2 | 2 | 2 | 2 | 2 | 1 | 2 | 0 | -- | -- | -- | -- |
| Forde PM et al.2018 ^[37]^; | 2 | 2 | 2 | 2 | 2 | 1 | 2 | 0 | -- | -- | -- | -- |
| Bott MJ et al.2018 ^[38]^ | 2 | 2 | 2 | 2 | 1 | 1 | 2 | 0 | -- | -- | -- | -- |
| Shen D et al, 2021 ^[39]^ | 2 | 2 | 2 | 2 | 2 | 1 | 2 | 0 | -- | -- | -- | -- |
| Eichhorn F et al, 2021 ^[40]^ | 2 | 2 | 2 | 2 | 2 | 2 | 1 | 0 | -- | -- | -- | -- |
| Bar, J., et al, 2021^[41]^ | 2 | 0 | 0 | 2 | 0 | 2 | 2 | 0 | -- | -- | -- | -- |
| Tong BC et al, 2021 ^[42]^ | 2 | 2 | 2 | 1 | 2 | 2 | 1 | 0 | -- | -- | -- | -- |
| Shu CA et al, 2020 ^[43]^ | 2 | 2 | 2 | 2 | 2 | 1 | 2 | 0 | -- | -- | -- | -- |
| Lee J et al, 2021 ^[44]^ | 2 | 2 | 2 | 2 | 2 | 1 | 1 | 0 | -- | -- | -- | -- |
| Zhang P et al, 2022^[45]^ | 2 | 2 | 2 | 2 | 2 | 2 | 2 | 0 | -- | -- | -- | -- |
| Gao S et al, 2020 ^[46]^ | 2 | 2 | 2 | 2 | 2 | 1 | 2 | 0 | -- | -- | -- | -- |
| Tao XL et al, 2020 ^[47]^ | 2 | 2 | 2 | 2 | 2 | 1 | 2 | 0 | -- | -- | -- | -- |
| Zhu, X et al, 2021^[48]^ | 2 | 1 | 1 | 2 | 0 | 0 | 0 | 0 | -- | -- | -- | -- |
| Wu YL et al, 2022 ^[49]^ | 2 | 2 | 2 | 2 | 2 | 2 | 2 | 2 | -- | -- | -- | -- |
| Zhao ZR et al, 2021 ^[50]^ | 2 | 2 | 2 | 2 | 2 | 1 | 2 | 0 | -- | -- | -- | -- |
| Hong MH et al, 2021 ^[51]^ | 2 | 2 | 0 | 2 | 2 | 1 | 2 | 0 | -- | -- | -- | -- |
| Rothschild SI et al, 2021^[52]^ | 2 | 2 | 2 | 2 | 2 | 1 | 2 | 0 | -- | -- | -- | -- |
| Tfayli A et al, 2020 ^[53]^ | 2 | 2 | 2 | 2 | 2 | 1 | 2 | 0 | -- | -- | -- | -- |
| Deng H et al, 2021 ^[54]^ | 2 | 2 | 0 | 2 | 2 | 2 | 2 | 0 | -- | -- | -- | -- |
| Shi, L., et al, 2021^[55]^ | 2 | 0 | 0 | 2 | 0 | 0 | 0 | 0 | -- | -- | -- | -- |
| Hong T et al, 2021 ^[56]^ | 2 | 2 | 0 | 2 | 2 | 2 | 2 | 0 | -- | -- | -- | -- |
| Hu Y et al, 2021 ^[57]^ | 2 | 2 | 0 | 2 | 2 | 2 | 2 | 0 | -- | -- | -- | -- |
| Cheng X. et al, 2022^[58]^ | 2 | 2 | 0 | 2 | 2 | 2 | 2 | 2 | -- | -- | -- | -- |
| Chen T et al, 2021 ^[59]^ | 2 | 2 | 0 | 2 | 2 | 2 | 2 | 0 | -- | -- | -- | -- |
| Wu J et al, 2022^[60]^ | 2 | 2 | 0 | 2 | 2 | 2 | 1 | 0 | -- | -- | -- | -- |
| Zhang, Y et al, 2021^[61]^ | 2 | 0 | 1 | 2 | 0 | 0 | 0 | 0 | -- | -- | -- | -- |
| Chen Y et al, 2021 ^[62]^ | 2 | 2 | 0 | 2 | 2 | 1 | 2 | 0 | -- | -- | -- | -- |
| Zhai H et al, 2022 ^[63]^ | 2 | 2 | 0 | 2 | 2 | 1 | 1 | 0 | -- | -- | -- | -- |
| Yao Y et al, 2022 ^[64]^ | 2 | 2 | 2 | 2 | 2 | 2 | 2 | 0 | -- | -- | -- | -- |
| Fan BS et al,2022 ^[65]^ | 2 | 2 | 2 | 2 | 2 | 2 | 2 | 1 | -- | -- | -- | -- |

*The items are scored 0 (not reported), 1 (reported but inadequate) or 2 (reported and adequate). The global ideal score being 16 for non-comparative studies and 24 for comparative studies.
