# Supplementary material for: Does major pathological response after neoadjuvant Immunotherapy in resectable nonsmall-cell lung cancers predict prognosis? A systematic review and meta-analysis
Source: Int J Surg. 2023 May 26;109(9):2794–807. doi: 10.1097/JS9.0000000000000496 (PMC10498860; doi:10.1097/JS9.0000000000000496)
Supplement: SUPPLEMENTARY MATERIAL [file js9-109-2794-s011.docx]

#install.packages(c("metafor","meta"))

library(metafor)

library(meta)

setwd("")

dat=read.table("3.txt",sep="\t",header=T,row.names=1,check.names=F)

ies.logit=escalc(xi=cases,ni=total,measure="PAS",data=dat)

pes.logit=rma(yi,vi,data=ies.logit,method="REML", test="knha")

pes.forest=metaprop(cases,total,authoryear,data=dat,sm="PAS",method.tau="REML",method.ci ="NAsm",incr=0.5,allincr=FALSE,addincr=FALSE,hakn=TRUE)

pdf("MPR .pdf",width=18,height=13)

pes.forest$event <- round(pes.forest$event)

forest(pes.forest, xlim = c(0,100), pscale =100, comb.fixed=FALSE,

comb.random = TRUE, lty.fixed=0, lty.random=2, type.study="square",

type.random="diamond", ff.axis="bold.italic", ff.fixed="bold.italic", ff.random="bold.italic",

ff.xlab="bold",

hetlab = "Heterogeneity:",

seTE.predict=TRUE,

rightcols=c("effect", "ci"),

rightlabs = c("MPR (%)", "95% CI"),

leftcols = c("studlab","Histology","Stage","dose", "IO", "event","n"),

leftlabs = c("Study","Histology","Stage","Treatment protocol","IO type", "Event cases","Sample size"), xlab = "MPR (%)",

fs.xlab=14,

fs.study=12,

fs.study.lables=12,

prediction = TRUE,

fs.heading=12,

squaresize = 0.5, col.square="navy", col.square.lines="navy", col.diamond="navy",

col.diamond.lines="navy",

print.Q=TRUE,

print.pval.Q=TRUE,

print.I2=TRUE,

print.I2.ci = TRUE,

print.tau2=TRUE,

print.tau2.ci = TRUE,

calcwidth.fixed = TRUE,

print.tau = TRUE,

print.tau.ci=TRUE,

calcwidth.pooled = TRUE,

calcwidth.hetstat = TRUE,

weight.study = "random",

just = "center",

col.by="grey",

digits=1,

digits.tau2=2,

digits.tau=2, colgap.forest.left = "0.5inch")

dev.off()

#Funnel plot and tests: http://www.metafor-project.org/doku.php/plots

pdf(".pdf",width=5,height=5)

funnel(pes.logit)

dev.off()

ranktest(pes.logit)

regtest(pes.logit)
